# PSMA PET in Imaging Prostate Cancer

**DOI:** 10.3389/fonc.2022.831429

**Published:** 2022-01-28

**Authors:** Ioannis Tsechelidis, Alexis Vrachimis

**Affiliations:** ^1^ Department of Nuclear Medicine, German Oncology Center, University Hospital of the European University, Limassol, Cyprus; ^2^ Cancer Research and Innovation Center (CARIC), Limassol, Cyprus

**Keywords:** staging, restaging, biochemical failure (BF), theranostics, PET/CT

## Abstract

After prostate malignancy diagnosis, precise determination of disease extent are fundamental steps for tailored made therapy. The earlier the diagnosis of the burden of the disease, the longer the survival in many cases. National and international guidelines are based on “classic” imaging technics combining radiological and nuclear medicine scans like CT, MRI and bone scintigraphy (BS). The most recent nuclear medicine development is the prostate specific membrane antigen (PSMA) PET and is emerging as the most promising tool of medical imaging, gaining ground every day. Nevertheless, the different onset among multiple studies fails to establish a worldwide admission and incorporation of this technique in guidelines and its position in workaday medical algorithms. It seems that the medical community agrees not to utilize PSMA PET for low-risk patients; intense debate and research is ongoing for its utility in intermediate risk patients. Contrariwise, in high-risk patients PSMA PET is confirmed outperforming CT and BS combined. Additionally, irrespectively to their castration status, patients with biochemical failure should be referred for PSMA PET. Even though PSMA PET reveals more extended disease than expected or exonerates equivalent lesions, thus impacting treatment optimization. Studies being in progress and future trials with clarify whether PSMA PET will be the new gold standard technic for specific groups of patients.

## Introduction

Prostatic cancer (PC) is the most common male malignancy worldwide and the third most common with regards of mortality (1). After its diagnosis, precise determination of extent of the disease and follow-up scheduling are fundamental steps for both, therapy framing and prognosis. The number, the nature and the sites of lesions offer the basis for a personalized treatment. The earlier the diagnosis of the burden of the disease, the longer the survival of many patients.

Conventional imaging techniques like CT and MRI face significant restriction as they fail to detect lymph nodal lesions measuring smaller than 8 mm (2). It is not uncommon for MRI, more precisely for DWI, to be unable to differentiate infiltrated from non-metastatic lymph nodes and consequently, lymph nodal secondaries are missed, especially in high-risk and intermediate-risk patients (3). Furthermore, CT and MRI often fail to correctly identify non-PC lymphadenopathies. Similarly, BS is in many cases not specific enough with regards to bone findings in PC patients. In all these cases, PSMA PET(/CT) eventuates to better delineate or/and clarify the status of the disease.

There is a highly emerging field on PSMA tracers labeled with different radioisotopes either for diagnostic, or for therapeutic indent. Multiple clinical studies have been performed and much more are in currently ongoing.

## Spectrum of PET Radiopharmaca in PC

18F-FDG remains the workhorse in PET imaging, however with very restricted applications for PC, as its role is limited in cases of neuroendocrine differentiation or of progressive disease presenting such characteristics. This occurs because of low to no glucose consumption by the classic prostatic primary and thereafter by its spread. Thus, the need for specific targeting arose.

Up to recently Choline labeled tracers either by 11C or 18F, were used as promising markers. This radiotracer, initially applied to restaging after biochemical recurrence and staging of high-risk patients has been practically abandoned due its low sensitivity (4). Other tracers like 11C -acetate or 18F-uorocyclobutane-1-carboxylic acid had been tested without standing up to the expectations presenting equal characteristics to Choline. Similarly, 18F-Uciclovire met the same fate to previously referred tracers (5).

**Figure 1:**
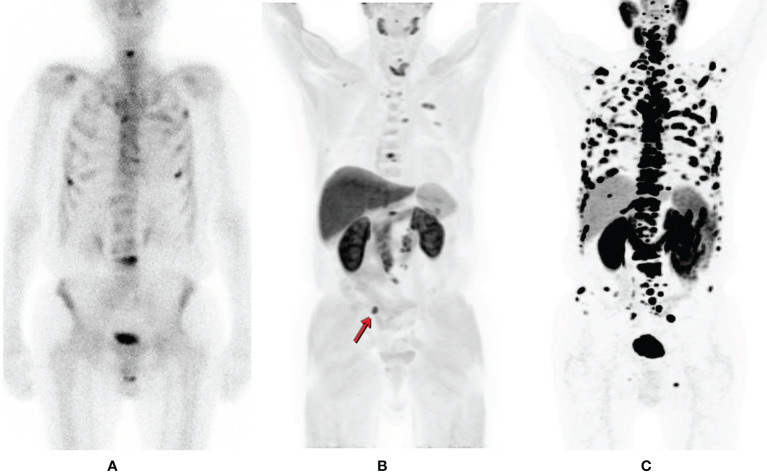
Patient suffering from prostate cancer is referred for bone scintigraphy after biochemical failure, detecting multiple sites of osteoblastic activity corresponding to multiple bone lesions **(A)**. Within one week the patient also underwent a 11C-Choline PET/CT in order to exclude visceral spread **(B)** for treatment planning. Apart from additional bone secondaries, a common iliac lymph nodal lesion right (arrow) is detected (maximum intensity projection (MIP); **(B)**. However, the true spread of the disease is much more extended by 68Ga-PSMA PET (MIP); **(C)** [republished with permission of the Hellenic Journal of Nuclear Medicine (7)].

Up to now, Prostate-specific membrane antigen (PSMA) seems to reign over all other tracers in PET PC imaging. PSMA also known as glutamate carboxypeptidase II, is a transmembrane glycoprotein highly expressed in prostate cancer cells. PSMA expression tends to increase with increased pathological Gleason grade and is thought to be upregulated with the emergence of androgen independence (6). An example of a PC patient imaged at the same time period with various tracers is illustrated in **Figure 1**.

## PSMA Ligands

Up to date several 68Ga ligands have been developed to target PSMA, e.g. PSMA-11 (known as HBED-CC or HBED-PSMA), PSMA-I&T and PSMA-617. A 68Ge/68Ga generator with T1/2 of 271 days is therefore widely used (8). However, in sites with cyclotron access PSMA-1007 seems like a major breakthrough as it shows an advantage over the 68Ga labelled PSMA tracers (9).

**Figure 2:**
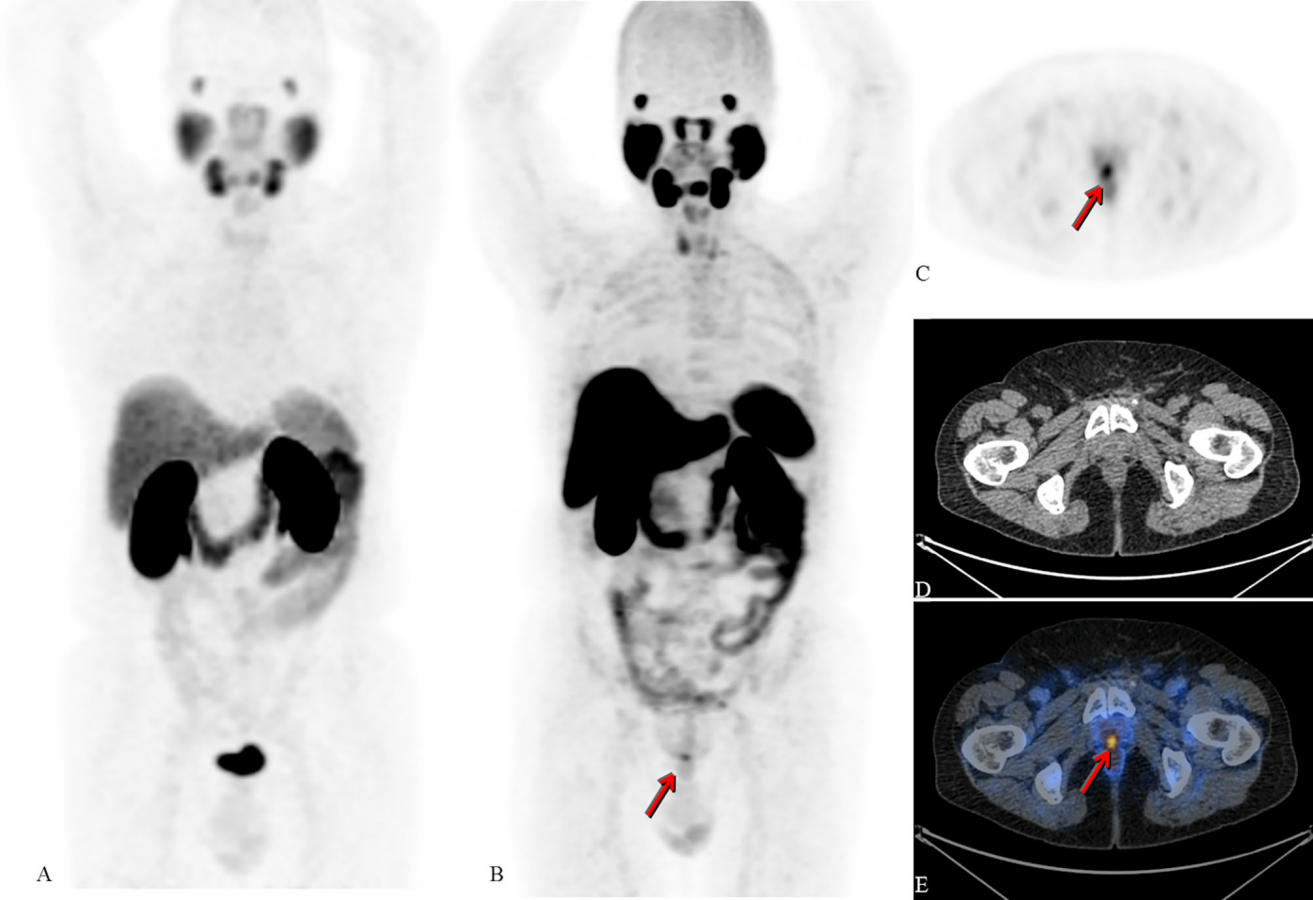
Radical prostatectomy in 2004 due to adenocarcinoma. Salvage radiotherapy due to biochemical recurrence in 2007 with negative restaging on conventional imaging. In 2013, new PSA rise without lesions on restaging with CT, MRI and bone scintigraphy; intermittent androgen-deprivation therapy. Patient referred for 68Ga-PSMA-11 PET/CT in 2016 (not available) and 2017 (maximum intensity projection (MIP); **(A)** negative for PSMA-expressing lesions (PSA levels at 0.8 and 1.0ng/mL, respectively). Due to further increase of PSA (1.1ng/mL) an 18F-PSMA-1007 PET/CT examination took place in 2018. Images B - E show 18F-PSMA -1007 PET/CT of the same patient **(B)**: MIP, **(C)**: axial cross section PET, **(D)**: axial cross section CT, **(E)**: fused axial PET/CT image). Arrows show unequivocal PSMA-expression corresponding to local recurrence; the quality of the image is not biased by PSMA-detection in the ureters or inside the bladder (in comparison to **A**) [republished with permission of the Hellenic Journal of Nuclear Medicine (7)].

Generally, todays’ tendency is the use of 18F labeled ligands rather than 68Ga labeled molecules because of the favorable isotope characteristics and imaging properties:

- Both, qualitative effect on image and quantification parameters could be positively influenced by superior positron energy (633keV vs. 68Ga, 1,899keV) and inferior positron yield of 18F (96.9% vs. 68Ga, 89,1).
- The diverse characteristics of the various 68Ge/68Ga generators make the already intricate procedure of combining the radioisotope to PSMA-molecule even more complex. Moreover, special enactments and legislations between connecting countries become an extra factor of distribution deceleration.
- Searching for a financially efficient process, high number of cases is required mainly because of the short T1/2 of 68Ga.
- The longer the T1/2, the better the image quality based on the advantage of time prolongation between the injection time and the onset of the imaging achieving an image with less noise and better tumor-to-background ratio.
- Though negligible at first sight, the heterogeneity in pharmacokinetic features privileges 18F-PSMA-1007 over 68Ga-PSMA-11, e.g., increased renal elimination of 68Ga-PSMA-11 over 18F-PSMA-1007, could lead to false negative results because a vital lesion of the prostate or prostatic bed could be masked by the presence of urine and so tracer accumulation in the urinary bladder (**Figure 2**).

## Staging

According to the Appropriate Use Criteria (AUC) for PSMA PET, reported in SNMMI PSMA PET is indicated for new diagnosis of PC of unfavorable intermediate and high-risk/very high-risk patients as staging, particularly if CT and BS show none to 5 distant lesions. On the other side, if extended metastatic burden has already been confirmed, there is no indication for PSMA PET as it will not change treatment (apart from identification of PSMA target in case that a radioligand therapy is striven). Furthermore, there is no clear evidence for PSMA PET in staging PC with Gleason < 7 (10). Approaching the cases of CRPC, more than half of them is staged M1 on PSMA PET from M0 on conventional imaging. Should 0 to 3-5 distant lesions be detected on CT and BS, PSMA PET could be of value to prove the patient’s oligometastatic status, so EBRT will be meaningfully applied.

Ferraro et al. retrospective analysis refers to 116 patients suffering from intermediate or high-risk PC having been investigated by 68Ga-PSMA-11 PET/CT or MRI on initial staging. Only 3 cases had no pathological PSMA expression of the primary malignancy (2,6%). LN spread was found in 28 cases (24%) and osseous secondaries in 14 patients (12%). 68Ga-PSMA-11 PET/CT leaded to upstaging of patients in 42/116 cases, meaning 36% of them, who had a therapy replanning (e.g., radiotherapy methods) facing lesions that were ignored by either clinical staging or conventional imaging (11, 12).

Multiple studies have shown that PSMA PET/CT has a moderate sensitivity but very high specificity for detection of nodal metastasis in intermediate-to-high-risk prostate cancer:

- Koerber and colleagues concluded that PSMA PET/CT is an excellent tool for lymphogenous spread on initial staging. PSMA-expressing LN metastases were detected in 90/280 men (32.1%) [>1/3 in extrapelvic lymph nodal groups (13)].
- Luiting et al. published a review with 11 studies (12) concerning sensitivity and specificity for N on initial staging:
- ✓ two prospective studies with 63 patients had per patient sensitivity of 64-100% and specificity 90%- 95% and per node sensitivity of 50-58% and specificity 96%- 100%
- ✓ nine retrospective studies with 696 cases had a per patient sensitivity of 33-100% and specificity of 80%- 100% and per node sensitivity of 24-96% and specificity of 98%-100%
- Petersen and Zacho published a systematic review for primary lymph node staging of intermediate and high-risk prostate cancer with 18 clinical trials and 969 patients with extended heterogeneity in terms of radiopharmaceuticals, kind of equipment, and risk status of patients (14); sensitivity ranged from 23 to 100%, specificity 67%-100%, positive predictive value 20%-100%, and negative predictive value 41%-100%. Overall, PSMA PET/CT demonstrated a better sensitivity and specificity compared to conventional imaging.
- The same group reported almost 1000 cases of non-low-risk PC patients prior to any treatment were included in an expedited systematic review that showed very encouraging results concerning diagnostic accuracy of PSMA PET for N status; different radioisotopes were included in the review for PSMA labelling and imaging either with PET/CT or PET/MRI (14).
- Thomas A. Hope at all published a multicentre prospective imaging trial including 764 cases of intermediate to high-risk PC who underwent 68Ga-PSMA-11-PET before prostatectomy and lymphadenectomy; the sensitivity and specificity of the exam was 0,40 and 0,95 respectively. According to this trial, a PSMA PET/CT without evidence of lymphogenous spread doesn’t mean that a surgeon will abandon lymphadenectomy. On the other hand, PSMA+ve nodes mean that N+ disease is a fact despite the rare cases of false positive scan (15).

PSMA PET/CT plays crucial role for distant lesions detection and for clarification of suspicious or undetermined lesions on CT or bone scan. Should a primary prostatic cancer show PSMA-expression, a PSMA+ and PSMA- finding will be characterized as secondary lesion or treated as unimportant/benign lesion respectively in the fragment of an imaging technic with high negative predictive value (16).

Roach et al. published the results of an Australian prospective multicenter study counting 431 patients with intermediate or high-risk patients referred for initial staging. The therapeutic strategy was revised in 21% of patients due to lesions revealed by 68Ga-PSMA-11 PET/CT scan (17).

A multicentre, two-arm, randomized study from Hofma et al. in Australia, analyzed 302 high-risk PC patients that had been randomly stratificated in two categories for staging either with CT and BS or 68Ga-PSMA-11 PET/CT. In this, 68Ga-PSMA-11 PET/CT was superior to the combination of CT and BS in all levels assessed; accuracy for N/M (92% vs 65%), sensitivity (85% vs 38%) and specificity (98% vs 91%). Treatment modification was performed in 27% of patients having undergone 68Ga-PSMA-11 PET/CT over 5% of the alternatives. Equivocal lesions were more often on CT and BS (23%) than in 68Ga-PSMA-11 PET/CT (7%). Furthermore, more than half of patients having been staged by CT and BS had higher radiation exposure (19,2 mSv) than those investigated by 68Ga-PSMA-11 PET/CT (8,4 mSv) (18).

This is most probably going to replace the 2018 consensus of the multidisciplinary experts (Focus 1) recommending CT and BS as indispensable imaging for advanced PC indifferently to castration status (19). Notwithstanding, 11 of 21 members of that panel, at the last modified Delphi process, replayed suggesting replacing conventional imaging by PSMA PET/CT. ION the same meeting, a consensus was reached that PSMA PET/CT should be indicated in staging only in selected cased of low-risk patients.

Driven from the high cost of the PSMA imaging the identification of those patients to have a clinical impact from the examination is needed. A group of Italian experts highlight the medical community the approx. 10% of the cases of patients that show no PSMA-expression, proposing as key of the problematic the correlation of PSMA-expression to immunochemistry which is unfortunately not available in every center, and it is time consuming. Moreover, recognizing the superiority of mpMRI for local evaluation of the disease, they suggest that PSMA PET/MRI could be the solution as one single whole-body exam. Finally, based on the ProPSMA results, they recommend the update of the current guidelines promoting PSMA PET/CT (20).

A systematic review from Awenat et al. assessed the value of 18F-PSMA-1007 PET/CT in PC staging using data from 8 studies, including 369 patients with Gleason scores range from 6 to 10. Overall, the sensitivity of 18F-PSMA-1007 PET/CT ranged from 74% to 100% on a per patient-based analysis and from 71% to 100% on a per lesion-based analysis; the specificity of 18F-PSMA-1007 PET/CT ranged from 76% to 100% on a per patient-based analysis and from 91% to 100% on a per lesion-based analysis; the accuracy of 18F-PSMA-1007 PET/CT ranged from 80% to 100% on a per patient-based analysis and from 93% to 95% on a per lesion-based analysis (9).

The recently published multicentre randomized study proPSMA study evaluated 68Ga-PSMA PET/CT in initial assessment of high-risk patients suffering from PC as compared to CT and BS. The superiority of 68Ga-PSMA PET/CT was proved and it was combined to inferior radiation exposure and associated with reduced unclear findings (18).

Almost 1000 cases of non-low-risk PC patients prior to any treatment were included in an expedited systematic review that showed very encouraging results concerning diagnostic accuracy of PSMA PET for N status; different radioisotopes were included in the review for PSMA labelling and imaging either with PET/CT or PET/MRI (14).

18F-DCFPyL is a further PSMA based PET ligand and in a study including more than 400 patients with PC of all Gleason scores achieved an excellent performance in staging with a detection rate of nearly 90% for PSA ≥ 0.5ng/ml and about 50% for lower PSA values (21).

Non-PC-related PSMA-uptake is in limited cases a headache also in the most experienced eyes, as a large list of different kind of malignant and nonmalignant conditions have been reported in the literature to experience PSMA uptake such as: bone related conditions (e.g. osteomyelitis, fracture, Paget’s disease, hemangioma, fibrous dysplasia, osteochondroma, polycythemia rubra vera etc.), inflammatory and infectious processes (e.g. sarcoidosis, tuberculosis, diverticulosis, amyloidosis od seminal vesicles, nodular fasciitis etc.), benign tumors (e.g. meningioma, thyroid and parathyroid adenomas, elastofibroma dorsi, thymoma, angiolipoma, pancreatic serous cystadenoma, desmoid tumors, myxoma, fibromatosis etc.) and other malignanies (e.g. glioma, head and neck SCC, thyroid cancer, hepatocellular carcinoma, cholangiocarcinoma, renal cancer, pancreatic cancer, neuroendocrine tumors, GIST, gynecological malignancies, osteosarcoma, melanoma, multiple myeloma etc.) (22, 23). However, in many cases characteristics such as topography, distribution, intensity of PSMA Uptake and morphology could help in avoiding pitfalls in most of the cases.

## Restaging/Biochemical Failure

Data derived from literature are more enthusiastic for PSMA PET/CT in case of restaging after biochemical relapse following radical therapies (e.g., prostatectomy, EBRT, brachytherapy etc.). According to a review the positive predictive value of 68Ga-PSMA-11 PET/CT in patients with PSA increase before salvage lymphadenectomy was 70-100%. These results were correlated to the PSA level after prostatectomy; detection rate was 11.3%-50.0% for PSA <0.2ng/mL, 20.0%- 72.7% for PSA 0.2-0.49ng/mL and 25.0%-87.5% for PSA 0.5 to <1.0 ng/mL (12).

In another prospective study, 68Ga-PSMA-11 PET/CT changed the therapeutic planning in 62% of cases with biochemical relapse after prostatectomy by detecting local relapse in 27%, lymphogenous spread in 39% and distant metastases in 16% of the cases (17).

In a clinical study of almost 280 patients with biochemical relapse after prostatectomy and two successive PSA measurement of ≥ 0.2ng/ml, 68Ga-PSMA PET/CT led to management change with an overall clinical impact even in low PSA values (42.4% for PSA 0.2-0.4ng/ml; 27.7% for PSA 0.5-1 ng/ml; 21.2% for 1.1-2ng/ml and 8.7% for PSA> 2ng/ml) (24).Müller et al. suggested that 68Ga-PSMA-11 PET/CT plays a significant role as it leads to changing the therapy planning of a significant number of patients with PSA increase (25). Concordant to other studies (12) it was proved that 68Ga-PSMA-11 PET/CT revealed correlative in half of patients with PSA inferior to 0,5 ng/ml. Furthermore, more than half of patients had a change of the treatment strategy; more specifically systemic therapy was reduced (from 60% to 34%), while emphasis was given to metastasis-dedicated plans almost one third of cases were referred to radiation treatment and only a patients’ minority of combination of radiotherapy and hormonotherapy with almost 50% of patients achieving complete response in half a year. Sonni et al. evaluating the results of nearly 300 cases also proved the major impact of 68Ga-PSMA-11 PET/CT to the management of patients suffering from PC either leading to acquittal of suspicious lesions in 1/3 of cases or revealing unknown metastases in 38% of them (26). A recently metaanalysis indicated that detection rate of 18F-PSMA-1007 PET/CT in patients with biochemical relapse is similar to 68Ga-PSMA-11 PET/CT (27).

These results are aligned with the recommendations of Focus 1 meeting according to which most members of the committee propound that PSMA PET/CT should replace CT and BS in patients without castration presenting PSA<0,5ng/ml post prostatectomy (28). However, no concordance was noticed between the panelist team on PSA cutoff with almost half of them presenting against a cutoff.

An interesting study by Ferraro et al. focused on negative PSMA PET/CT in PC patients with biochemical relapse. After evaluating 120 scans, this study showed that if an immunohistologically confirmed primary PC didn’t overexpress PSMA on baseline exam it was unlikely to detect PSMA-expressing lesions on restaging irrelevant to PSA level or ADT effect. More precisely, more than half of the patients had a negative PSMA PET/CT scan even after significantly elevated PSA (29).

De Reijke et al. performed a study of 95 patients presenting biochemical failure having initially undergone Radical Prostatectomy (RP) or External Beam Radiotherapy (EBRT). Their data state that the topographical positions and the quantity of pathological spots on 68Ga-PSMA PET/CT led to management change in PC patients and that their survival rates could be prognosticated. Furthermore, the doubling time of PSA showed a positive correlation to 68Ga-PSMA PET/CT. The key finding concerning the prognostic effect of 68Ga-PSMA PET/CT was that, patients presenting biochemical relapse and having negative scan showed a longer progression free survival as compared to those with a positive scan (30).

Additionally, Hoffmann et al. demonstrated that the localisation of tumor burden was positively correlated with the PSA levels as patients with higher PSA level was more probable to have positive lymph nodal or distant lesions on 68Ga-PSMA PET/CT as compared to patients with lower PSA level which were more probable to have a positive local recurrence (18).

A study elaborated by Fourquet et al. studied almost 300 patients with PC without distant spread on conventional imaging with PSA rise after definitive local therapy that showed a progressive increase of PSA after two successive assessments. In this study 68Ga-PSMA PET/CT achieved excellent performance detecting secondary deposits of PC and impelled treatment planification change in 2/3 of cases. The performance rate of the imaging was higher with PSA>1ng/ml, particularly for abdominopelvic LN spread (31).

A meta-analysis compared Choline to PSMA as ligands for detection rate PET/CT scan in patients after PSA rise. PSMA outperformed Choline (54% vs 27%) in cases of PSA ≤1 ng/ml. Nevertheless, no obvious advantage is given to PSMA when PSA>1ng/ml (32). On the other hand, Choline and Fluciclovine demonstrated inferior diagnostic performance to any labelled PSMA molecule. The best, proportionate outcome was note between PSMA-11, PSMA-1007, PSMAI&T, and DCFPyl in a systematic review and network meta-analysis (33).

In a further study, 68Ga-PSMA-11 PET/CT and multiparametric MRI (mpMRI) were compared with regards to local relapse and lymph nodal spread in biochemical failure PSA> 0,2ng/ml. mpMRI was superior to 68Ga-PSMA-11 PET/CT for identifying local relapse, while inverse results are exported for lymphogenous or distant spread (34).

PSMA PET is also indicated if cases of incomplete biochemical response after prostatectomy. In case of RTx of the primary, no worldwide accepted definition of biochemical recurrence exists because non-infiltrated prostatic tissue is still detected. Furthermore, and due to the fact that patients undergoing radical radiotherapy are mainly low risk individuals it remains unclear whether PET PSMA is of value as first line imaging procedure (10).

Till now immunohistochemistry is not available in each center and it is time consuming. Thus, nomograms to predict positive PSMA PET/CT may help clinical to choose PSMA especially for restaging PC. In one of the earliest attempts a prediction nomogram for positive 68Ga-PSMA-11 PET/CT in patients with raised PSA up to 1ng/ml after radical prostatectomy was proposed by Rausher et al. However, even the cases without risk factors (PSA, ongoing ADT, ISUP grade) had only less than 50% possibility of a positive scan (35) The same nomogram was further evaluated externally by Bianchi et al. with more than 400 patients. In this validation, ADT was proved less crucial than PSA in comparison to the original model. However, PSA and ongoing ADT are estimated as positive predictive factors in a nomogram cut-off 35% (36) From these data it was evident that the proposed nomogram suffered from suboptimal accuracy and improved formulas were needed to provide a more trustworthy and accurate model to clinicians to avoid false negative PSMA PET/CT. In this direction the Italian group suggested a novel prediction nomogram for 68 Ga-PSMA-11 PET/CT for patients with biochemical relapse after radical prostatectomy. Using this, significantly improved rates were observed: 40.3% for patients with first biochemical recurrence; 54.0% for biochemical relapse after salvage therapy; 60.5% after radical prostatectomy and 86.9% in advanced stage prostate cancer before second line of systemic treatment. This nomogram had a cut-off value 40% in positive scan prediction, while the real clinical benefit was >10% according to Decision Curve Analysis. Furthermore, ISUP grade, PSA, PSA doubling time, and clinical setting were independent predictors of a positive scan in a multivariate analysis (37). Besides false negative, also a low rate (<10%) of false positive 68Ga-PSMA-11 PET/CT scans is recently reported in a prospective multicenter trial counting 635 patients with biochemical relapse after radiotherapy. In most cases, the false positivity of the examination was attributed to post-radiotherapy prostate uptake (38).

## Influence of Androgen Deprivation Therapy (ADT) on PSMA-Ligand PET/CT Imaging of PC

Vaz et al. attempted to investigate the impact of ADT on PSMA-expression (39). In spite of the rareness of PSMA PET/CT in cases being treated by ADT and the absence of consensus for such an indication, short term (e.g., 6-months) ADT seems to enhance the intensity of pathological PSMA-expression. Contrariwise, long term ADT (e.g., 3 years) reduces the uptake of the tracer, maybe also due to treatment response and corresponding restriction of the lesions’ extent and higher possibility for partial volume effects. Hence, the European Association of Urology guidelines, recommended proceeding with PSMA PET/CT when a patient’s PSA gets over 0.2 ng/ml as restaging, optimally prior to ADT onset. In the opposite direction, taking in consideration the reinforcement of PSMA-expression by short-term ADT, the sensitivity of PSMA PET/CT could be increased in cases of biochemical recurrence with PSA inferior to 0.5 ng/ml. It is imperative that further work is needed to reach to global consensus of the medical community on this topic.

## Reporting System

Conventional imaging technics have been well established methods in national health care systems and are treated by experts with confidence. Nowadays, the more PSMA PET/CT gets endorsed in international guidelines, standardized reporting systems are developing in order to reduce gray zone results, and thus enhancing the embracement of the modality. Two promising systems have been published, both requiring validation of their effectiveness and further evolution:

- Reporting System for Prostate-Specific Membrane Antigen- Targeted PET Imaging: PSMA-RADS Version 1.0 (40) and the
- Prostate Cancer Molecular Imaging Standardized Evaluation (PROMISE): Proposed miTNM Classification for the Interpretation of PSMA-Ligand PET/CT (41).

Moreover, radiomic features are an evolving part of the PSMA PET/CT imaging field, claiming better delineation of sites of primary malignancy, correct Gleason scoring and lymph nodal assessment (42). Nonetheless, further work is needed.

## Impediments to Clinical Applications

Apart from the benefits coming out from PSMA PET/CT for patients suffering from PC, several restrictions are noted. First, PET cameras are not always available as they are missing from nuclear medicine departments particularly of the remote regions for various reasons. Endorsement of PSMA in international guidelines is needed warranted (43). Another crucial point is the clarification of its role for the patient’s course after upstaging his status. More work is needed to correlate the management change of a patient with survival benefit (17).

In conclusion, PSMA PET/CT has shown unchallengeable results in PC both in initial setting and biochemical failure. Irrespectively of PSA level, PSMA PET/CT outperforms CT and BS combined.

## Author Contributions

IT and AV concepted and designed the review, collected, and analyzed the representative studies from the literature, wrote and approved the manuscript.

## Conflict of Interest

The authors declare that the research was conducted in the absence of any commercial or financial relationships that could be construed as a potential conflict of interest.

## Publisher’s Note

All claims expressed in this article are solely those of the authors and do not necessarily represent those of their affiliated organizations, or those of the publisher, the editors and the reviewers. Any product that may be evaluated in this article, or claim that may be made by its manufacturer, is not guaranteed or endorsed by the publisher.
